# *catena*-Poly[bis(1,3-benzo­thia­zol-3-ium) [[di­chlorido­anti­monate(III)]-di-μ-chlorido-μ-oxido-[chlorido­anti­monate(III)]-μ-chlorido]]

**DOI:** 10.1107/S2056989016000785

**Published:** 2016-01-20

**Authors:** Oussama Chebout, Mhamed Boudraa, Sofiane Bouacida, Hocine Merazig, Chaouki Boudaren

**Affiliations:** a Unité de Recherche de Chimie de l’Environnement et Moléculaire Structurale, CHEMS, Université Frères Montouri Constantine, 25000, Algeria; b Département Sciences de la Matière, Faculté des Sciences Exactes et Sciences de la Nature et de la Vie, Université Oum El Bouaghi, Algeria

**Keywords:** crystal structure, organic–inorganic hybrid compound, anti­mony, hydrogen bonding, π–π stacking

## Abstract

The title compound, {(C_7_H_6_NS)_2_[Sb_2_Cl_6_O]}_*n*_, contains two benzo­thia­zolidium cations and one tri-μ-chlorido-tri­chlorido-μ-oxido-di­anti­monate(III) anion. The structure of the inorganic cation may be described as as being built up from two polyhedra, *i.e.* a square-pyramidal SbCl_4_O and a distorted octa­hedral SbOCl_5_ unit, sharing a common face (comprising the O atom and two Cl atoms). The two benzo­thia­zole cations are quasi-planar and subtend a dihedral angle of 19.93 (5)°. The crystal packing can be described by alternating (100) layers and [001] chains of the organic cations and inorganic anions connected through an extensive three-dimensional network of N—H⋯Cl, C—H⋯O and C—H⋯Cl hydrogen bonds. This is consolidated by slipped π–π stacking, with centroid-to-centroid distances between the benzo­thia­zole rings of 3.7111 (18)–3.8452 (16) Å. These inter­actions link the mol­ecules within the layers and also link the layers together and reinforce the cohesion of the ionic structure.

## Abstract 2

In the crystal, alternating layers and chains of the organic cations and inorganic anions are connected through an extensive three-dimensional network of N—H⋯Cl and C—H⋯Cl hydrogen bonds.π–π stacking inter­actions link the mol­ecules within the layers and also link the layers together and reinforce the cohesion of the ionic structure.

## Chemical context   

The coordination chemistry of anti­mony has both a practical and theoretical inter­est (Abboud *et al.*, 2007 ▸; Bujak & Angel, 2006 ▸). Recently, the use of anti­mony complexes in cancer chemotherapy has become a topic of inter­est (Demicheli *et al.*, 2006 ▸; Rais *et al.*, 2000 ▸). As part of our ongoing studies of benzo­thia­zole-based coordination networks (Bouchareb *et al.*, 2014 ▸), we now report the polymeric structure of new organic–inorganic hybrid compound {(C_7_H_6_NS)_2_[Sb_2_Cl_6_O]}_*n*_, (I).
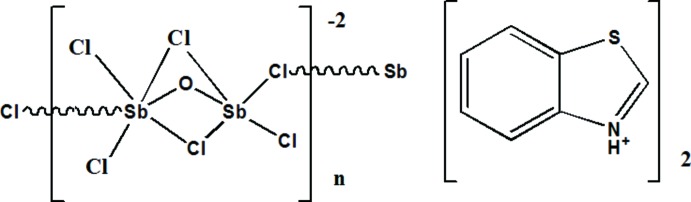


## Structural commentary   

**Figure 1:**
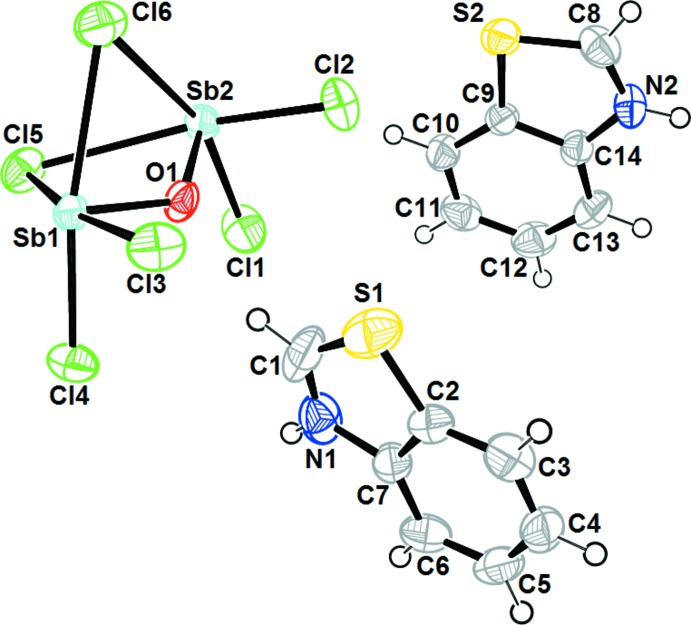
The asymmetric unit of (I), showing the atom-numbering scheme. Displacement ellipsoids are drawn at the 50% probability level.

The title compound contains two benzo­thia­zolidium cations and one tri-μ-chlorido-tri­chlorido-μ-oxido-di­anti­monate(III) anion (Sb_2_Cl_6_O^2−^). The mol­ecular geometry and the atom-numbering scheme are shown in Fig. 1 ▸.

**Figure 2:**
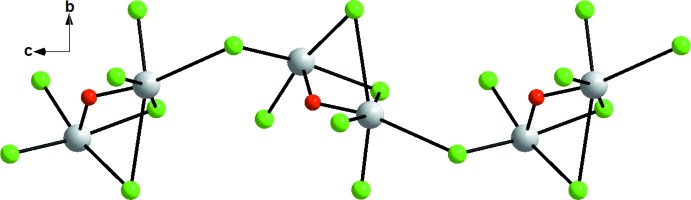
View of a polymeric chain of Sb_2_Cl_6_O along the *c* axis.

The structure of the inorganic anion may be described as two polyhedra, square-pyramidal SbCl_4_O and distorted octa­hedral SbOCl_5_, sharing a common face (O1, Cl5 and Cl6). In the first polyhedron, four Cl atoms (Cl3–Cl4–Cl5–Cl6) form a basal plane with the Sb1 atom lying 0.3011 (2) Å below the plane. The apical position is occupied by the O1 atom. In the second polyhedron, the O1 atom occupies the apical position and four Cl atoms (Cl1–Cl2–Cl5–Cl6) form the base equatorial plane with Sb2 displaced by 0.4168 (1) Å from it. The geometry of the Sb2 atom can be described as distorted octa­hedral, a sixth coordination is observed at a longer distance, with Sb2 coordinated by the adjacent Cl3^i^ atom at a distance of 3.546 (4) Å [symmetry code: (i) 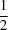 − *x*, 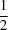 + *y*, 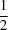 − *z*], forming an infinite chain parallel to [001] (Fig. 2 ▸). This distance is significantly shorter than the sum of the relevant van der Waals radii of 4.01 Å (*r*Sb = 2.1 Å and *r*Cl = 1.91 Å) and in good agreement with those found in [SbCl_3_(C_25_H_22_O_2_P_2_)] (Razak *et al.*, 1999 ▸) and in [(CH_3_)_2_NH(CH_2_)_2_NH_3_][SbCl_5_] (Bujak & Angel, 2006 ▸). In this mol­ecule, the angle between the two equatorial planes is 75.86 (2)°.

The Sb—O bridge distances of 1.9404 (16) and 1.9460 (17) Å are similar to those found in the Sb_2_Cl_6_O_2_ moiety (Abboud *et al.*, 2007 ▸). Excluding the longest bond (Sb2—Cl3^i^), the terminal Sb—Cl bonds are in the range 2.3974 (8)–2.4982 (8) Å and are shorter than the bridging bonds [2.7522 (8)–3.3244 (9) Å] and are in good agreement with those found in C_26_H_28_N_8_O_6_Sb_4_Cl_10_ (Abboud *et al.*, 2007 ▸). However, the Sb—O—Sb bond angle is 123.56 (9)° which is very different to that observed in Cs_2_Sb_2_O_2_(OH)_8_ (Mikhaylov *et al.*, 2011 ▸) and the Sb_2_Cl_6_O_2_ moiety (Abboud *et al.*, 2007 ▸). The dihedral angle between the mean planes of the two benzo­thia­zole cations is 19.93 (5)°.

## Supra­molecular features   

**Figure 3:**
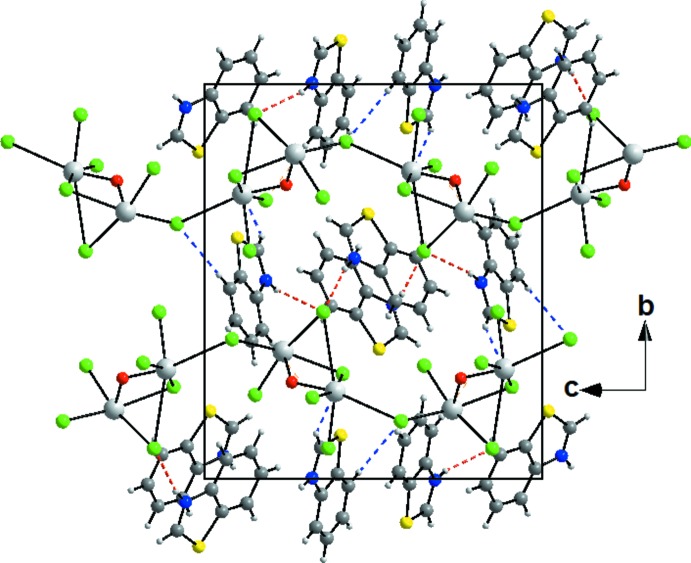
Part of diagram packing of the title compound, viewed along the *a* axis, showing alternating chains and layers connected by N—H⋯Cl and C—H⋯Cl hydrogen bonds (shown as dashed lines).

The crystal packing can be described by alternating (100) layers and [001] chains of organic cations and inorganic anions connected through an extensive network of N—H⋯Cl, C—H⋯O and C—H⋯Cl hydrogen bonds, leading to the formation of a three-dimensional network (Table 1 ▸, Fig. 3 ▸). The packing is consolidated by slipped π–π stacking with centroid-to-centroid distances of 3.7111 (18)–3.8452 (16) Å between the benzo­thia­zole rings. These inter­actions link the mol­ecules within the layers and also link the layers together, reinforcing the cohesion of the ionic structure.

## Synthesis and crystallization   

A solution of SbCl_3_ (45.6 mg, 0.2 mmol) in water (10 ml) was added dropwise to a solution of benzo­thia­zole (0.5 ml, 4.6 mmol) in ethanol (10 ml). The mixture was then refluxed with stirring for 3 h and the resulting solution was left to stand at room temperature. Colorless crystals were obtained after several days.

## Refinement   

Crystal data, data collection and structure refinement details are summarized in Table 2 ▸. Approximate positions for all H atoms were first obtained from the difference electron density map. However, the H atoms were placed into idealized positions and refined using the riding-atom approximation. The applied constraints were: C—H = 0.93 Å and N—H = 0.86 Å, *U*_iso_ = 1.2*U*_eq_(C or N).

## Supplementary Material

Crystal structure: contains datablock(s) I. DOI: 10.1107/S2056989016000785/hg5468sup1.cif

Structure factors: contains datablock(s) I. DOI: 10.1107/S2056989016000785/hg5468Isup2.hkl

CCDC reference: 1447413

Additional supporting information:  crystallographic information; 3D view; checkCIF report

## Figures and Tables

**Table 1:** Hydrogen-bond geometry (Å, °)

*D*—H⋯*A*	*D*—H	H⋯*A*	*D*⋯*A*	*D*—H⋯*A*
N1—H1*N*⋯Cl6^i^	0.86	2.37	3.200 (3)	162
N2—H2*N*⋯Cl6^ii^	0.86	2.35	3.145 (3)	153
C1—H1⋯O1	0.93	2.27	3.152 (4)	159
C8—H8⋯Cl5^iii^	0.93	2.72	3.327 (3)	124
C10—H10⋯Cl3^iv^	0.93	2.78	3.612 (3)	150
C13—H13⋯Cl2^ii^	0.93	2.76	3.524 (3)	140

Symmetry codes: (i) 
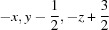
; (ii) 
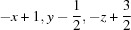
; (iii) 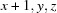; (iv) 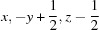.

**Table 2:** Experimental details

Crystal data	
Chemical formula	(C_7_H_6_NS)_2_[Sb_2_Cl_6_O]
*M*_r_	744.58
Crystal system, space group	Monoclinic, *P*2_1_/*c*
Temperature (K)	295
*a*, *b*, *c* (Å)	10.2826 (2), 16.2448 (3), 14.9849 (3)
β (°)	111.674 (1)
*V* (Å^3^)	2326.09 (8)
*Z*	4
Radiation type	Mo *K*α
μ (mm^−1^)	3.20
Crystal size (mm)	0.17 × 0.13 × 0.11

Data collection	
Diffractometer	Bruker APEXII CCD
Absorption correction	Multi-scan (*SADABS*; Sheldrick, 2002 ▸)
*T*_min_, *T*_max_	0.630, 0.746
No. of measured, independent and observed [*I* > 2σ(*I*)] reflections	20349, 5344, 4627
*R*_int_	0.026
(sin θ/λ)_max_ (Å^−1^)	0.651

Refinement	
*R*[*F* ^2^ > 2σ(*F* ^2^)], *wR*(*F* ^2^), *S*	0.022, 0.050, 1.02
No. of reflections	5344
No. of parameters	244
H-atom treatment	H-atom parameters constrained
Δρ_max_, Δρ_min_ (e Å^−3^)	0.54, −0.77

Computer programs: *APEX2* and *SAINT* (Bruker, 2011 ▸), *SIR2002* (Burla *et al.*, 2005 ▸), *SHELXL97* (Sheldrick, 2008 ▸), *ORTEP-3 for Windows* and *WinGX* (Farrugia, 2012 ▸) and *DIAMOND* (Brandenburg & Berndt, 2001 ▸).

## supplementary crystallographic information

### Crystal data

**Table:**

(C_7_H_6_NS)_2_[Sb_2_Cl_6_O]	*F*(000) = 1416
*M**_r_* = 744.58	*D*_x_ = 2.126 Mg m^−^^3^
Monoclinic, *P*2_1_/*c*	Mo *K*α radiation, λ = 0.71073 Å
Hall symbol: -P 2ybc	Cell parameters from 8790 reflections
*a* = 10.2826 (2) Å	θ = 2.5–27.5°
*b* = 16.2448 (3) Å	µ = 3.20 mm^−^^1^
*c* = 14.9849 (3) Å	*T* = 295 K
β = 111.674 (1)°	Block, colorless
*V* = 2326.09 (8) Å^3^	0.17 × 0.13 × 0.11 mm
*Z* = 4	

### Data collection

**Table:**

Bruker APEXII CCD diffractometer	5344 independent reflections
Radiation source: sealed tube	4627 reflections with *I* > 2σ(*I*)
Graphite monochromator	*R*_int_ = 0.026
φ and ω scans	θ_max_ = 27.5°, θ_min_ = 2.9°
Absorption correction: multi-scan (*SADABS*; Sheldrick, 2002)	*h* = −11→13
*T*_min_ = 0.630, *T*_max_ = 0.746	*k* = −19→21
20349 measured reflections	*l* = −19→18

### Refinement

**Table:**

Refinement on *F*^2^	Primary atom site location: structure-invariant direct methods
Least-squares matrix: full	Secondary atom site location: difference Fourier map
*R*[*F*^2^ > 2σ(*F*^2^)] = 0.022	Hydrogen site location: inferred from neighbouring sites
*wR*(*F*^2^) = 0.050	H-atom parameters constrained
*S* = 1.02	*w* = 1/[σ^2^(*F*_o_^2^) + (0.0186*P*)^2^ + 1.5448*P*] where *P* = (*F*_o_^2^ + 2*F*_c_^2^)/3
5344 reflections	(Δ/σ)_max_ = 0.001
244 parameters	Δρ_max_ = 0.54 e Å^−^^3^
0 restraints	Δρ_min_ = −0.77 e Å^−^^3^

### Special details

**Table:**

Geometry. All e.s.d.'s (except the e.s.d. in the dihedral angle between two l.s. planes) are estimated using the full covariance matrix. The cell e.s.d.'s are taken into account individually in the estimation of e.s.d.'s in distances, angles and torsion angles; correlations between e.s.d.'s in cell parameters are only used when they are defined by crystal symmetry. An approximate (isotropic) treatment of cell e.s.d.'s is used for estimating e.s.d.'s involving l.s. planes.
Refinement. Refinement of *F*^2^ against ALL reflections. The weighted *R*-factor *wR* and goodness of fit *S* are based on *F*^2^, conventional *R*-factors *R* are based on *F*, with *F* set to zero for negative *F*^2^. The threshold expression of *F*^2^ > σ(*F*^2^) is used only for calculating *R*-factors(gt) *etc*. and is not relevant to the choice of reflections for refinement. *R*-factors based on *F*^2^ are statistically about twice as large as those based on *F*, and *R*- factors based on ALL data will be even larger.

### Fractional atomic coordinates and isotropic or equivalent isotropic displacement parameters (Å^2^)

**Table:**

	*x*	*y*	*z*	*U*_iso_*/*U*_eq_	
S1	0.36748 (12)	0.17148 (5)	0.98344 (7)	0.0662 (3)	
N1	0.1904 (3)	0.05865 (18)	0.94373 (19)	0.0539 (7)	
H1N	0.114	0.0319	0.9156	0.065*	
C1	0.2113 (4)	0.1303 (2)	0.9189 (2)	0.0653 (10)	
H1	0.1448	0.1582	0.8683	0.078*	
C2	0.4125 (3)	0.08232 (16)	1.05140 (19)	0.0386 (6)	
C3	0.5374 (3)	0.0621 (2)	1.1261 (2)	0.0509 (8)	
H3	0.612	0.0988	1.147	0.061*	
C4	0.5459 (3)	−0.0141 (2)	1.1676 (2)	0.0528 (8)	
H4	0.6282	−0.0293	1.2172	0.063*	
C5	0.4350 (4)	−0.0684 (2)	1.1374 (2)	0.0516 (8)	
H5	0.4439	−0.1191	1.1678	0.062*	
C6	0.3131 (3)	−0.04978 (18)	1.0645 (2)	0.0474 (7)	
H6	0.2391	−0.087	1.0443	0.057*	
C7	0.3030 (3)	0.02653 (17)	1.02135 (19)	0.0372 (6)	
S2	0.52690 (8)	0.11063 (4)	0.59703 (6)	0.04448 (17)	
N2	0.7036 (2)	0.00265 (15)	0.68158 (18)	0.0436 (6)	
H2N	0.7828	−0.0203	0.7127	0.052*	
C8	0.6923 (3)	0.07956 (18)	0.6561 (2)	0.0465 (7)	
H8	0.769	0.1143	0.6695	0.056*	
C9	0.4653 (3)	0.01241 (16)	0.60657 (18)	0.0339 (6)	
C10	0.3292 (3)	−0.01737 (19)	0.5748 (2)	0.0431 (7)	
H10	0.2533	0.0166	0.5433	0.052*	
C11	0.3111 (3)	−0.0985 (2)	0.5917 (2)	0.0501 (7)	
H11	0.2208	−0.1199	0.5712	0.06*	
C12	0.4237 (3)	−0.15006 (19)	0.6385 (2)	0.0530 (8)	
H12	0.4069	−0.205	0.6482	0.064*	
C13	0.5579 (3)	−0.12215 (18)	0.6705 (2)	0.0481 (7)	
H13	0.6331	−0.1567	0.7016	0.058*	
C14	0.5770 (3)	−0.03964 (16)	0.65440 (19)	0.0359 (6)	
Sb1	−0.067085 (18)	0.323213 (10)	0.767483 (12)	0.03143 (5)	
Sb2	0.053422 (18)	0.221298 (11)	0.613337 (12)	0.03397 (5)	
Cl1	0.01992 (9)	0.07748 (5)	0.63368 (7)	0.0589 (2)	
Cl2	0.30403 (7)	0.20616 (5)	0.68094 (6)	0.05062 (18)	
Cl3	0.13613 (8)	0.35386 (5)	0.91846 (5)	0.05065 (18)	
Cl4	−0.14276 (8)	0.21318 (5)	0.84967 (6)	0.05007 (18)	
Cl5	−0.24438 (8)	0.26668 (4)	0.59142 (6)	0.04931 (18)	
O1	0.05721 (19)	0.24501 (12)	0.74157 (12)	0.0391 (4)	
Cl6	0.04721 (8)	0.42304 (5)	0.64881 (6)	0.0558 (2)	

### Atomic displacement parameters (Å^2^)

**Table:**

	*U*^11^	*U*^22^	*U*^33^	*U*^12^	*U*^13^	*U*^23^
S1	0.0921 (7)	0.0379 (4)	0.0703 (6)	0.0017 (4)	0.0319 (5)	0.0061 (4)
N1	0.0376 (13)	0.0731 (19)	0.0455 (15)	0.0001 (13)	0.0092 (12)	−0.0065 (14)
C1	0.073 (2)	0.071 (2)	0.0456 (19)	0.042 (2)	0.0148 (18)	0.0111 (17)
C2	0.0497 (16)	0.0334 (13)	0.0350 (15)	−0.0018 (12)	0.0181 (13)	−0.0021 (11)
C3	0.0474 (17)	0.062 (2)	0.0414 (17)	−0.0141 (15)	0.0139 (14)	−0.0112 (15)
C4	0.0485 (18)	0.076 (2)	0.0313 (15)	0.0127 (17)	0.0116 (14)	0.0045 (15)
C5	0.070 (2)	0.0477 (17)	0.0422 (17)	0.0085 (16)	0.0267 (17)	0.0122 (14)
C6	0.0581 (19)	0.0430 (16)	0.0458 (17)	−0.0131 (14)	0.0247 (15)	−0.0014 (13)
C7	0.0363 (14)	0.0440 (15)	0.0308 (14)	0.0019 (12)	0.0117 (12)	−0.0024 (11)
S2	0.0533 (4)	0.0327 (3)	0.0566 (5)	0.0085 (3)	0.0310 (4)	0.0065 (3)
N2	0.0318 (12)	0.0456 (13)	0.0510 (15)	0.0069 (11)	0.0125 (11)	−0.0009 (11)
C8	0.0454 (17)	0.0419 (16)	0.060 (2)	−0.0037 (13)	0.0282 (15)	−0.0071 (14)
C9	0.0385 (14)	0.0333 (13)	0.0329 (14)	0.0054 (11)	0.0168 (12)	0.0027 (11)
C10	0.0368 (15)	0.0549 (18)	0.0368 (15)	0.0077 (13)	0.0125 (12)	0.0067 (13)
C11	0.0422 (16)	0.061 (2)	0.0444 (17)	−0.0152 (15)	0.0135 (14)	−0.0025 (15)
C12	0.062 (2)	0.0387 (16)	0.058 (2)	−0.0107 (15)	0.0214 (17)	0.0041 (14)
C13	0.0533 (18)	0.0384 (15)	0.0495 (18)	0.0097 (14)	0.0153 (15)	0.0106 (13)
C14	0.0344 (13)	0.0375 (14)	0.0357 (14)	0.0054 (11)	0.0127 (12)	0.0011 (11)
Sb1	0.03409 (9)	0.02825 (9)	0.03458 (10)	0.00560 (7)	0.01575 (7)	−0.00018 (7)
Sb2	0.03473 (10)	0.04105 (10)	0.02678 (9)	0.00516 (8)	0.01211 (7)	−0.00064 (7)
Cl1	0.0568 (5)	0.0425 (4)	0.0830 (6)	−0.0050 (4)	0.0325 (4)	−0.0093 (4)
Cl2	0.0351 (4)	0.0611 (5)	0.0567 (5)	0.0025 (3)	0.0181 (3)	−0.0138 (4)
Cl3	0.0601 (5)	0.0487 (4)	0.0381 (4)	−0.0131 (4)	0.0122 (3)	−0.0065 (3)
Cl4	0.0570 (4)	0.0486 (4)	0.0529 (4)	−0.0093 (3)	0.0301 (4)	0.0031 (3)
Cl5	0.0457 (4)	0.0430 (4)	0.0491 (4)	−0.0024 (3)	0.0057 (3)	0.0020 (3)
O1	0.0460 (11)	0.0463 (11)	0.0271 (9)	0.0225 (9)	0.0160 (8)	0.0044 (8)
Cl6	0.0466 (4)	0.0579 (5)	0.0518 (5)	−0.0107 (4)	0.0051 (4)	0.0103 (4)

### Geometric parameters (Å, º)

**Table:**

S1—C1	1.678 (4)	C8—H8	0.93
S1—C2	1.732 (3)	C9—C10	1.388 (4)
N1—C1	1.265 (4)	C9—C14	1.393 (3)
N1—C7	1.404 (4)	C10—C11	1.368 (4)
N1—H1N	0.8599	C10—H10	0.93
C1—H1	0.93	C11—C12	1.391 (4)
C2—C7	1.385 (4)	C11—H11	0.93
C2—C3	1.396 (4)	C12—C13	1.360 (4)
C3—C4	1.373 (5)	C12—H12	0.93
C3—H3	0.93	C13—C14	1.389 (4)
C4—C5	1.379 (4)	C13—H13	0.93
C4—H4	0.93	Sb1—O1	1.9404 (16)
C5—C6	1.358 (4)	Sb1—Cl4	2.4545 (7)
C5—H5	0.93	Sb1—Cl3	2.4982 (8)
C6—C7	1.384 (4)	Sb1—Cl5	2.7522 (8)
C6—H6	0.93	Sb1—Cl6	2.9524 (8)
S2—C8	1.679 (3)	Sb2—O1	1.9460 (17)
S2—C9	1.742 (3)	Sb2—Cl1	2.3974 (8)
N2—C8	1.299 (4)	Sb2—Cl2	2.4081 (7)
N2—C14	1.392 (3)	Sb2—Cl5	3.0473 (8)
N2—H2N	0.8599	Sb2—Cl6	3.3244 (9)
			
C1—S1—C2	89.81 (16)	C9—C10—H10	121.2
C1—N1—C7	114.1 (3)	C10—C11—C12	121.9 (3)
C1—N1—H1N	123	C10—C11—H11	119.1
C7—N1—H1N	122.9	C12—C11—H11	119.1
N1—C1—S1	115.3 (3)	C13—C12—C11	121.6 (3)
N1—C1—H1	122.3	C13—C12—H12	119.2
S1—C1—H1	122.3	C11—C12—H12	119.2
C7—C2—C3	120.2 (3)	C12—C13—C14	116.8 (3)
C7—C2—S1	110.3 (2)	C12—C13—H13	121.6
C3—C2—S1	129.4 (2)	C14—C13—H13	121.6
C4—C3—C2	117.5 (3)	C13—C14—N2	127.1 (3)
C4—C3—H3	121.3	C13—C14—C9	122.1 (3)
C2—C3—H3	121.3	N2—C14—C9	110.8 (2)
C3—C4—C5	121.4 (3)	O1—Sb1—Cl4	88.74 (6)
C3—C4—H4	119.3	O1—Sb1—Cl3	85.37 (6)
C5—C4—H4	119.3	Cl4—Sb1—Cl3	90.28 (3)
C6—C5—C4	121.8 (3)	O1—Sb1—Cl5	80.90 (6)
C6—C5—H5	119.1	Cl4—Sb1—Cl5	91.00 (3)
C4—C5—H5	119.1	Cl3—Sb1—Cl5	166.17 (3)
C5—C6—C7	117.6 (3)	O1—Sb1—Cl6	78.52 (6)
C5—C6—H6	121.2	Cl4—Sb1—Cl6	166.56 (3)
C7—C6—H6	121.2	Cl3—Sb1—Cl6	92.87 (2)
C6—C7—C2	121.5 (3)	Cl5—Sb1—Cl6	82.88 (2)
C6—C7—N1	128.1 (3)	O1—Sb2—Cl1	91.07 (6)
C2—C7—N1	110.4 (3)	O1—Sb2—Cl2	88.67 (6)
C8—S2—C9	90.54 (14)	Cl1—Sb2—Cl2	91.64 (3)
C8—N2—C14	114.6 (2)	O1—Sb2—Cl5	73.22 (5)
C8—N2—H2N	122.7	Cl1—Sb2—Cl5	93.65 (2)
C14—N2—H2N	122.7	Cl2—Sb2—Cl5	161.21 (2)
N2—C8—S2	114.0 (2)	O1—Sb2—Cl6	69.00 (6)
N2—C8—H8	123	Cl1—Sb2—Cl6	158.15 (3)
S2—C8—H8	123	Cl2—Sb2—Cl6	96.56 (2)
C10—C9—C14	120.0 (2)	Cl5—Sb2—Cl6	72.59 (2)
C10—C9—S2	130.0 (2)	Sb1—Cl5—Sb2	72.175 (18)
C14—C9—S2	110.0 (2)	Sb1—O1—Sb2	123.56 (9)
C11—C10—C9	117.5 (3)	Sb1—Cl6—Sb2	65.814 (16)
C11—C10—H10	121.2		
			
C7—N1—C1—S1	−0.1 (4)	C8—N2—C14—C9	−1.1 (3)
C2—S1—C1—N1	0.8 (3)	C10—C9—C14—C13	1.2 (4)
C1—S1—C2—C7	−1.2 (2)	S2—C9—C14—C13	−178.9 (2)
C1—S1—C2—C3	178.1 (3)	C10—C9—C14—N2	−178.8 (2)
C7—C2—C3—C4	−0.2 (4)	S2—C9—C14—N2	1.1 (3)
S1—C2—C3—C4	−179.4 (2)	O1—Sb1—Cl5—Sb2	−17.75 (6)
C2—C3—C4—C5	−0.6 (5)	Cl4—Sb1—Cl5—Sb2	−106.31 (2)
C3—C4—C5—C6	1.0 (5)	Cl3—Sb1—Cl5—Sb2	−11.07 (11)
C4—C5—C6—C7	−0.5 (4)	Cl6—Sb1—Cl5—Sb2	61.70 (2)
C5—C6—C7—C2	−0.3 (4)	O1—Sb2—Cl5—Sb1	18.27 (6)
C5—C6—C7—N1	178.1 (3)	Cl1—Sb2—Cl5—Sb1	108.29 (3)
C3—C2—C7—C6	0.7 (4)	Cl2—Sb2—Cl5—Sb1	2.25 (9)
S1—C2—C7—C6	180.0 (2)	Cl6—Sb2—Cl5—Sb1	−54.403 (18)
C3—C2—C7—N1	−178.0 (3)	Cl4—Sb1—O1—Sb2	124.26 (11)
S1—C2—C7—N1	1.3 (3)	Cl3—Sb1—O1—Sb2	−145.35 (12)
C1—N1—C7—C6	−179.4 (3)	Cl5—Sb1—O1—Sb2	33.05 (11)
C1—N1—C7—C2	−0.8 (4)	Cl6—Sb1—O1—Sb2	−51.47 (11)
C14—N2—C8—S2	0.6 (3)	Cl1—Sb2—O1—Sb1	−124.01 (11)
C9—S2—C8—N2	0.1 (2)	Cl2—Sb2—O1—Sb1	144.37 (12)
C8—S2—C9—C10	179.2 (3)	Cl5—Sb2—O1—Sb1	−30.53 (10)
C8—S2—C9—C14	−0.7 (2)	Cl6—Sb2—O1—Sb1	46.83 (10)
C14—C9—C10—C11	−0.6 (4)	O1—Sb1—Cl6—Sb2	24.73 (6)
S2—C9—C10—C11	179.5 (2)	Cl4—Sb1—Cl6—Sb2	6.05 (11)
C9—C10—C11—C12	−0.1 (4)	Cl3—Sb1—Cl6—Sb2	109.41 (2)
C10—C11—C12—C13	0.4 (5)	Cl5—Sb1—Cl6—Sb2	−57.378 (18)
C11—C12—C13—C14	0.2 (5)	O1—Sb2—Cl6—Sb1	−25.97 (6)
C12—C13—C14—N2	179.1 (3)	Cl1—Sb2—Cl6—Sb1	−0.64 (7)
C12—C13—C14—C9	−1.0 (4)	Cl2—Sb2—Cl6—Sb1	−112.00 (2)
C8—N2—C14—C13	178.9 (3)	Cl5—Sb2—Cl6—Sb1	52.283 (18)

### Hydrogen-bond geometry (Å, º)

**Table:**

*D*—H···*A*	*D*—H	H···*A*	*D*···*A*	*D*—H···*A*
N1—H1*N*···Cl6^i^	0.86	2.37	3.200 (3)	162
N2—H2*N*···Cl6^ii^	0.86	2.35	3.145 (3)	153
C1—H1···O1	0.93	2.27	3.152 (4)	159
C8—H8···Cl5^iii^	0.93	2.72	3.327 (3)	124
C10—H10···Cl3^iv^	0.93	2.78	3.612 (3)	150
C13—H13···Cl2^ii^	0.93	2.76	3.524 (3)	140

Symmetry codes: (i) −*x*, *y*−1/2, −*z*+3/2; (ii) −*x*+1, *y*−1/2, −*z*+3/2; (iii) *x*+1, *y*, *z*; (iv) *x*, −*y*+1/2, *z*−1/2.
